# Preimplantation Flow Testing of Ahmed Glaucoma Valve and the Early Postoperative Clinical Outcome

**DOI:** 10.5005/jp-journals-10008-1128

**Published:** 2013-01-15

**Authors:** Emma Jones, Pouya Alaghband, Jason Cheng, Laura Beltran-Agullo, Kin Sheng Lim

**Affiliations:** Consultant, Department of Glaucoma, Accident and Emergency, Moorfields Hospital NHS Trust, London, United Kingdom; Glaucoma Research Fellow, Department of Ophthalmology, St Thomas’ Hospital, London, United Kingdom; Clinical Glaucoma Fellow, Department of Ophthalmology, St Thomas’ Hospital, London, United Kingdom; Clinical Glaucoma Fellow, Department of Ophthalmology, St Thomas’ Hospital, London, United Kingdom; Consultant Ophthalmic Surgeon,Department of Ophthalmology Glaucoma Lead, St Thomas’ Hospital, Westminster Bridge Road, London, SE1 7EH, United Kingdom

**Keywords:** Glaucoma, Aqueous shunt, Methods, Adverse effects, Ahmed implant.

## Abstract

**Purpose:** The Ahmed glaucoma valve (AGV) implant is designed to prevent early postoperative hypotony. There is evidence of variation in hypotony rates in clinical trials which may be due to surgical technique variation, entry site leakage or valve defects from ‘over priming'. We describe a simple preimplantation gravity driven test to assess valve function after priming that may reduce hypotony rates.

**Materials and methods:** Retrospective case note review. An *in vivo* flow test of AGVs, based on the gravity driven test was introduced prior to implantation. The onset and offset of flow through the valve was measured by altering the height of a bottle of balanced saline solution. We rejected the AGV, if there was fluid still flowing at 10 cm (7 mm Hg) or if there was no flow at 17 cm of water (12 mm Hg). The AGV implantation surgery was without mitomycin C, with a 25G needle entry tract, a corneal or scleral patch graft tube cover and without intracameral viscoelastic.

**Results:** Twenty Ahmed valves were implanted in 16 patients between July 2008 and October 2009. Test failure resulted in four AGV being rejected. The mean preoperative pressure was 29 mm Hg (range, 10-57 mm Hg) and the intraocular pressure (IOP) at 7 days postoperatively was 15 mm Hg (range, 3-52 mm Hg). Hypotony, defined as an IOP of less than 5 mm Hg on two consecutive assessments, was present in two eyes (10%).

**Conclusion:** *In vivo* flow testing is an important safety check for the AGV. There are also other mechanisms after implantation that can cause an unexpected high or low IOP.

**How to cite this article:** Jones E, Alaghband P, Cheng J, Beltran-Agullo L, Lim KS. Preimplantation Flow Testing of Ahmed Glaucoma Valve and the Early Postoperative Clinical Outcome. J Current Glau Prac 2013;7(1):1-5.

## INTRODUCTION

The Ahmed glaucoma valve (AGV) (New World Medical, Rancho Cucamonga, CA, USA) is recognized as the only commonly used glaucoma drainage device that can provide immediate intraocular pressure (IOP) control. It has an integral valve design with unidirectional flow that allows aqueous to flow when pressure reaches a level that opens the two opposing elastic membranes, which is called the ‘opening pressure', and when the pressure drops below a critical level the membranes of the valve close, called the ‘closing pressure'. The first generation of AGV (S1, S2, S3 models) had a silicone tube and silicone membrane valve, with a polypropylene plate. As polypropylene has been shown to be more inflammatory than silicone a new version AGV, model FP7, with a flexible silicone plate containing the silicone valve housed in a polypropylene box was introduced in 2003.^1^

The reliability of FP7 AGV valve has not been consistent in studies and an *in vitro* study reported that the closing pressure of half of the six FP7 AGV tested was less than 5 mm Hg.^2^ These valves are ineffective in controlling low flow and therefore at risk of causing hypotony if they had been implanted. Several commentaries mention the risk of damaging the valve mechanism from excessive force during priming, risking postoperative hypotony.^3,4^ Given the variation and unpredictability of the valves before and after priming, one method of ensuring a functioning, non-damaged valve is to perform preimplantation testing. We have adapted the gravity driven flow test described by Porter so that the valve could be tested in a simple and sterile manner during surgery.^5^

Our primary aim was to minimize the risk of hypotony in the early postoperative period by only implanting AGV valves, where we had established a closing mechanism. Our secondary aim was to improve IOP control in these eyes by implanting a tube whose valve had been shown to open above 12 mm Hg.

## MATERIALS AND METHODS

**Fig. 1:**
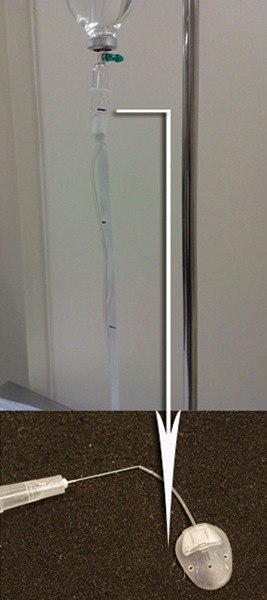
*In vivo* gravity flow test. Bottle of normal saline with giving set is attached to the AGV implant via a Rycroft cannula. The white arrow demonstrates the distance between the water meniscus of the giving set and the level of the AGV implant. This distance is the pressure in centimeters of water and can be converted to millimeters of mercury

A retrospective review of patients who underwent Ahmed glaucoma drainage device implantation after July 2008 was undertaken from our glaucoma surgery database. In St Thomas' Glaucoma Department, AGV are only implanted into eyes that are likely to have a low aqueous production rate, such as uveitic glaucoma, neovascular glaucoma and previous cyclodiode laser ablation treatment. A gravity driven flow test to measure the opening pressure and closing pressure of the AGV by onset and offset of flow across the valve has been described by Porter.^5^ We adapted this test by connecting a bottle of sterile balanced saline solution (BSS) via an infusion set to the valve and measuring the opening pressure and closing pressure by onset and offset of flow through the valve when altering the height of the BSS bottle. The test was carried out at the time of surgery after the AGV had been primed by BSS with a Rycroft cannula to produce a jet, as recommended by the manufacturer. The Rycroft cannula was left *in situ* and then connected to the BSS via a giving set (Fig. 1). The distance between the meniscus of the water in the infusion set chamber and the level at the tip of the Rycroft cannula gives a measurement of the pressure being applied to the valve, where a 13.6 cm height of water is equal to 10 mm Hg. The bottle is gradually raised until flow through the valve occurs and this height is recorded, then the bottle is lowered until it reaches a point where the water does not flow. We rejected the AGV, if there was no flow at 17 cm of water (12 mm Hg) or if fluid was still flowing at 10 cm (7 mm Hg).

Surgery was carried out after preparation with a single drop of adrenaline 1:1,000 and apraclonidine 1% (Alcon, UK). The margins of the quadrant were marked with ink at the limbus, a fornix-based conjunctival flap was created and no mitomycin C applied. The plate was sutured to the sclera with 7-0 prolene with a position 10 mm from the limbus and central to the bordering recti muscles. A paracentesis was created with a feather blade, and no intracameral viscoelastic was used. The tube was trimmed and passed through a scleral tunnel made by entry into the anterior chamber 2 mm behind the limbus with a 25G orange needle. The tube position was confirmed to be in the mid anterior chamber and not abutting the iris or cornea. The anterior chamber was then inflated with BSS via the paracentesis. The tunnel was checked to be watertight with fluorescein 2% (Minims, Chauvin Pharmaceuticals, UK), the exposed tube was covered with a corneal or scleral patch graft and then the conjunctiva was closed at the limbus.

## RESULTS

A total of 20 AGV FP7 were inserted in 16 patients. The clinical characteristics are shown Table 1. All of the patients after July 2008 had an AGV implanted that had passed our gravity driven flow test criteria. Four AGVs failed our testing criteria and were rejected, of which three were due to flow at below 10 cm of water height (7 mm Hg) and one due to no flow above 17 cm water height (12 mm Hg).

**Table 1:** **Table 1:** Demographics of the study population

		*Post-testing*	
Number of eyes (patients)		20 (16)	
Male:Female		9:7	
Age mean (range)		61 (29-82)	
Right:Left		10:10	
Ethnicity			
Caucasian		8	
Afro-Caribbean		6	
Indian		2	
Diagnosis			
Vein occlusion with secondary		5	
Rubeosis		5	
Proliferative diabetic retinopathy		1	
Uveitis		3	
POAG		1	
PACG		1	
Congenital rubella with		0	
microphthalmos		2	
Ocular ischemia			
Aphakic glaucoma		1	
Complicated cataract surgery		1	
ICCE			
Secondary anterior chamber IOL			

The IOP measurements before and after the implantation of AGV are shown Table 2. The mean preoperative pressure was 29 mm Hg (range, 10-57 mm Hg) and was lowered by day 7 to 15 mm Hg (range, 3-52 mm Hg). Of the two eyes with hypotony (IOP < 5 mm Hg) despite AGV testing, one eye had a shallow AC with peripheral choroidal effusion at week one and resolved after one intracameral viscoelastic injection. The second hypotonous eye had ‘kissing' choroidal effusions due to leakage around the tube entry site with an anterior bleb. This tube was successfully resited at a second operation without further complication. There were 6 eyes with early postoperative IOP greater than 15 mm Hg at day 7 (range, 18-52 mm Hg). In two of these eyes, there was still a significant reduction of IOP compared to their preoperative pressure, with a reduction from 57 to 20 mm Hg and 34 to 25 mm Hg respectively. Not one of these six patients had virgin conjunctiva; five had previous cyclodiode laser treatment two had trabeculectomy and two had tube surgery.

The visual acuity was less than or equal to 6/60 in 16 eyes prior to tube placement and in 14 eyes postsurgery. Two eyes had an improvement of two Snellen lines of acuity and one eye lost two Snellen lines of acuity.

**Table 2:** **Table 2:** IOP measurement before and after AGV FP7 implant

Mean IOP mm Hg prior to operation (range)		29 (10-57)	
Mean IOP mm Hg at day 1 (range)		11 (2-40)	
Mean IOP mm Hg at 7 days postopenings (range)		15 (3-52)	
Hypotony (IOP < 5 mm Hg) at day 1		2	

**Table 3:** **Table 3:** Comparison of reported rates of hypotony after surgery with Ahmed drainage devices^1,6,8,9,15-17^

*Study*		*Design*		*Follow-up (months)*		*Hypotony*		*Choroidal effusion*		*Shallow anterior chamber*		*Strategies to avoid hypotony*	
Jones 2012 Present study		Prospective single center 20 AGV FP7		3		Hypotony 2/20 (10%)		2/10 (10%)		1/20 (5%)		Preimplantation on device testing	
Christakis AVB 2011		Prospective multicenter 124 AGV FP7		12		NA		16 (13%)		18 (15%)		Viscoelastic in anterior chamber	
Budenz ABC 2010		Prospective multicenter 143 AGV FP7 Excluded previous cyclodiode		12		Hypotony maculopathy 5 (3%)		21(15%)		27 (19%)		Viscoelastic permitted at surgeons discretion	
Hinckle 2007		Retrospective 26 eyes AGV S2 25 eyes AGV FP7		>12		Not stated		3 (12%) S2 5 (20%) FP7				Unknown	
Ishida 2006		Prospective, multicenter 66 eyes AGV S2 66 eyes AGV FP7 Excluded previous cyclodiode		6-30		2 (3%) S2 2 (3%) FP7		4 (6%) S2 4 (6%) FP7		5 (8%) S2 2 (3%) FP7		Unknown	
Huang 1999		Retrospective multicenter 159 eyes S2				13/159 (8%)		10 (6%)		13/159 (8%)		Unknown	
Ayyala 1997		Retrospective 85 eyes AGV S2		>6		8/85 (9.4%)		1/85		6/85 (7%)		Unknown	
Coleman 1995		Prospective multicenter 60 eyes AGV S2		>12		8/60 (13%)		13/60 (22%)		2/60		Unknown	

## DISCUSSION

The Ahmed has a number of advantages compared to the nonvalved Baerveldt tube, in that it is relatively easy to implant, does not require manipulation of the recti muscles and it has the potential for a lower initial IOP which may provide a better long-term clinical outcome.^6,7^

The Ahmed implant's Venturi-based, flow restrictive mechanism is designed to open between pressure of 8 to 10 mm Hg and prevent hypotony and its complications. However, in clinical studies there have been varying reports of hypotony, shallow anterior chamber and visual threatening maculopathy and choroidal detachments ranging from 1 to 20% (Table 3).^6,8,9^ In addition, there appears to be variation among the different AGV implants. The incidence of hypotony requiring surgical intervention may be higher for the Ahmed FP7 compared to the Ahmed S2.^1^

This variation in early postoperative hypotony may be due to a number of factors, including leakage at the tube entry site, aqueous shut down, variation in surgical technique and also valve mechanism defects. Moss and Trope noted that the force required to prime the valve is not consistent, and, in clinical practice, priming is often repeated more than once to produce a jet, which could contribute to changes valve resistance to flow.^2^ It is thought that ‘over priming' of the implant may damage the valve mechanism, essentially rendering the implant valveless and at risk of overdrainage.^3^ Our gravity driven flow test is a useful check for potential primary failure of the AGV that may occur during ‘over priming' or from manufacturing variation. The gravity testing we carried out was immediately after high pressure priming. From this testing, four out of 24 (17%) AGVs were rejected due to excess flow at low pressures or no flow at high pressures. Having rejected these implants, our hypo-tony and hypotony-associated complications are lower than expected. Our population was likely to have a low aqueous production rate as all of them had either cyclodiode laser, rubeosis or uveitis preoperatively, and therefore would be at higher risk of aqueous shutdown postsurgery. Most studies have previous cyclodiode as an exclusion criterion.^6,8^ In addition, other studies use or allow the use of viscoelastic in the anterior chamber^6,10^ or use a vicryl ligation suture^11^ to lower the risk of hypotony, potentially underestimating the true hypotony associated from defective valves.

However, the IOP we measured were not consistently replicated postoperatively, as low and high IOPs still occurred. There are several explanations for this as numerous variables influence our study population, testing of the valve, surgical technique and the postsurgical healing response. Iatrogenic damage to the valve mechanism from manual handling during surgery and variation in surgical technique, such as a ‘loose fitting' scleral tunnel with leakage around the tube may also occur, as it did in one of our hypotony cases. Therefore, careful examination for an anterior bleb is essential as tube revision may be necessary.

High IOP from increased resistance and reduced flow can result from the tight conjunctival and tenons adhesion surrounding the plates aided by plasmin and coagulative, or the tube being blocked by fibrin or blood. A recent investigation into the surface topography of the common glaucoma drainage devices found that the AGV S2 and AGV FP7 were significantly rougher than the Molteno and Baerveldt, which correlated with tenon fibroblast adhesion and may lead to excessive fibrovascular reactions in AGV and a higher rate of occurrence of postimplantation hypertensive phase and failure due to fibrous encapsulation.^10^ The scarring response in our patient group may also be expected to be brisker as they had undergone previous surgery or laser which may prime the conjunctiva.

One weakness of our study is the lack of a control group. It is our belief that a valve that fails the preimplantation flow test would increase the risk of an adverse outcome. It would therefore not be justified to use an implant that we feel is not functioning as we would expect. We have compared our data to other published studies (Table 3) and, as discussed, we feel our group had more risk factors for hypotony and our protocol did not use intraoperative provisc or tube ligature. This strengthens our view point that pre-implantation flow testing is important in preventing postoperative hypotony.

Numerous strategies had been developed to reduce flow through glaucoma aqueous shunts during the early postoperative period in order to reduce the risk of early postoperative hypotony and its associated compli-cations.^3,4,12-14^

The valve mechanism makes the AGV a sophisticated medical device and provides definite advantages for early IOP control if functioning, and our test may make the immediate postoperative IOP of the AGV more predictable.
